# Association between antenatal care follow-up and institutional delivery service utilization: analysis of 2016 Ethiopia demographic and health survey

**DOI:** 10.1186/s12889-019-7854-2

**Published:** 2019-11-07

**Authors:** Eskezaiw Abebe, Abdu Seid, Getnet Gedefaw, Zelalem T. Haile, Gillian Ice

**Affiliations:** 1 School of Midwifery, College of Health Sciences, Woldia University, Woldia, Ethiopia; 2 Department of Social Medicine, Ohio University Heritage, College of Osteopathic Medicine, Dublin, OH USA; 3 Department of Social Medicine, Ohio University Heritage, College of Osteopathic Medicine, Athens, OH USA

**Keywords:** Antenatal care, Institutional delivery, EDHS, Ethiopia

## Abstract

**Background:**

Globally, the magnitude of maternal mortality is the major public health problem. Nearly all (99%) of maternal deaths occur in low- and middle-income countries. Of which 66% occur in sub-Saharan Africa. Institutional delivery under the hygienic environment with the necessary skills and equipment promotes to identify and treat complications, infections, and the death of the mother and baby. In Ethiopia, the utilization of maternal health services is very low. For instance, 62% of women had antenatal care utilization during pregnancy while only 26% of women utilize institutions for delivery in 2016. Therefore, this study examined the association between antenatal care follow up and intestinal delivery among a nationally representative woman in Ethiopia.

**Methods:**

A cross-sectional study design was used to examine 7575 women from the 2016 Ethiopia Demographic and Health Survey. Both descriptive and inferential statistics were utilized. Variables in the bivariate logistic regression with *p*-value < 0.2 were entered into the multivariable logistic regression. Odds ratios and corresponding 95% confidence intervals (CI) were reported. In the multivariable analysis, variables with *p*-value < 0.05 were considered as statistically significant.

**Results:**

The prevalence of institutional delivery service utilization for last childbirth was 11.3%. In comparison with women with no antenatal care visits, the multivariable odds ratio (95% confidence interval) of institutional delivery among those who attend one to three and four or more antenatal care visit were 2.49 (1.66, 3.74) and 3.90 (2.60, 5.84), respectively. Other factors significantly associated with institutional delivery include urban residence 2.25 (1.44, 3.51), complete primary education 3.22 (2.09, 4.98), complete secondary or higher education 1.59 (1.16, 2.17), poorer household wealth index 2.57 (1.57, 4.20), middle household wealth index 1.63 (1.05, 2.52), and richer household wealth index 1.56(1.03, 2.58).

**Conclusion:**

Antenatal care follow-up was significantly associated with institutional delivery service utilization. As the number of antenatal care visits increased the odds of facility delivery increased. Thus, improved access and utilization of antenatal care can be an effective strategy to increase institutional deliveries and optimal maternal and child health outcomes.

## Background

Globally, maternal mortality has declined from 532,000 in 1990 to 303,000 in 2015 [1], but the magnitude of maternal mortality remains major public health problems. Every day, about 830 women die from a preventable cause related to pregnancy and childbirth complications in the world. Almost all (99%) of maternal deaths occur in low-and-middle-income countries, 66% of which in Sub-Saharan Africa alone [2, 3]. The 2016 Ethiopia Demographic and Health Survey (EDHS) reported that maternal mortality ratio (MMR) is estimated to be 412 deaths per 100,000 live births which was high among rural and non-institutional delivered women [4].

Most pregnancy and childbirth complications are early preventable or treatable. Postpartum hemorrhage, sepsis, severe preeclampsia/eclampsia, obstructed labor, and unsafe abortion are accounted for 75 % of maternal deaths. Maternal mortality can be reduced through universal access to antenatal care, skilled care during childbirth, and postnatal care [3]. Skilled birth attendants during childbirth in a hygienic environment with necessary skills and types of equipment are important to identify and treat complications, infections, and death of the mother, and child [5].

The burden of home delivery is not only limited to a maternal health problem, but it also ends up with perinatal and neonatal morbidity and mortality [6]. One of the key strategies for reducing maternal morbidity and mortality is increasing institutional delivery service utilization of pregnant women under the care of skilled birth attendants [7].

Despite the continuous effort to encourage institutional delivery, many Ethiopian women still give birth at home. Improving the quality of antenatal care services is likely to contribute to rapid increases in skilled birth attendance and better health outcomes for women and children [8]. In Ethiopia, 62% of women had ANC visits during pregnancy but only 26% of women gave birth in the health facility [4]. Therefore, understanding the association between antenatal care utilization and institutional delivery services is of paramount importance to identify the potential role that ANC utilization plays in promoting institutional delivery. We hypothesized that ANC service utilization would be positively associated with institutional delivery among Ethiopian women.

## Methods

### Data and sampling design

Data for the study was derived from the 2016 EDHS. In brief, the 2016 EDHS was designed to provide nationally representative data on various health indicators for the country as a whole, and nine regional states and two city administrations. The sample for the 2016 EDHS was a stratified two-stage probability sample selected in two stages. First, each region was stratified into urban and rural areas. Then a sample of clusters were selected using a probability proportional to the size and with independent selection in each sampling stratum. The resulting lists of households served as a sampling frame for the selection of households in the second stage. A fixed number of households per cluster were selected with an equal probability systematic selection from the newly created household listing. Finally, all women age 15–49 who were either permanent residents of the selected households or visitors who stayed in the household the night before the survey were eligible to be interviewed A detailed description study design and methods of data collection for the 2016 EDHS are available elsewhere [4].

**Fig. 1:**
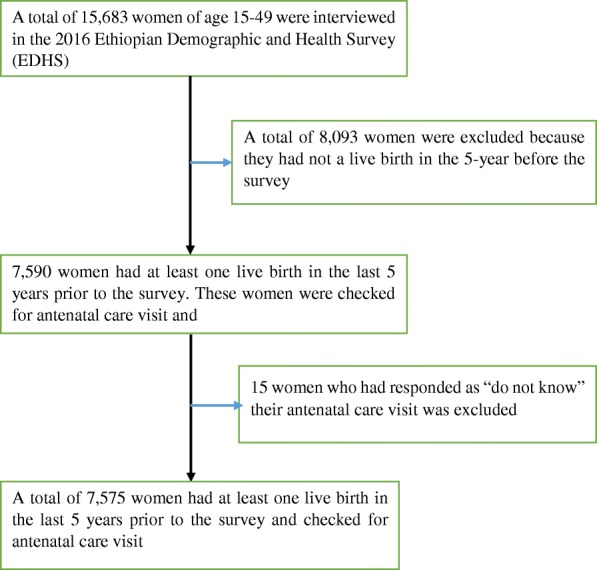
Schematic presentation of selecting sample from EDHS 2016 data

A total of 15,683 women aged 15–49 years were interviewed in the 2016 EDHS, of which 7590 women had at least one live birth in the last 5 years prior to the survey. Fifteen women who were responded “do not know” for the question related to antenatal care visits were excluded. This final analytic sample for the current study included 7575 participants (Fig. 1).

### Main outcome of interest

#### Institutional delivery

The main outcome of interest was place of delivery for a woman’s most recent birth. This variable was dummy coded so that respondents who reported giving birth at a health institution were coded as “yes”, otherwise “no”.

### Exposure measurement

#### Antenatal care (ANC)

Antenatal care follow-up was defined based on the self-reported frequency of any of ANC services provided by skilled health personnel in the health institution, and was categorized as “no history of ANC”, “one to three” and “four or above”.

### Covariates

Based on existing literature, sociodemographic, fertility-related and health facility-related characteristics were included as covariates.

#### Sociodemographic characteristics

This included maternal age at birth of the most recent child, religion, educational status, marital status, place of residence, household wealth index, exposure to media (read newspaper, listened to the radio, or watched television at least once a week, and classified to ‘exposed’ and ‘non-exposed’).

#### Fertility-related characteristics

This included birth order of the most recent child categorized as “first”, “2–4”, or “five or above”, and “wantedness” of the pregnancy of the recent child categorized as “wanted then”, “wanted later”, and “wanted no more”.

#### Facility-related characteristics

This included the mother’s perceived distance of the nearby health facility, categorized as “big problem” or “not a big problem”.

### Statistical analysis

Data were analysed using SPSS version 20 statistical software [9]. Sampling weight was applied for all analysis procedures to account for complex survey design and unequal probabilities of selection. Frequencies and proportions were used to describe the characteristics of the study sample. Rao-Scott chi-square test that adjusts for complex sample design was used to examine the bivariate associations between each covariate and institutional delivery. Both bivariate and multivariable logistic regression analysis were performed to identify the association between ANC and institutional delivery service utilization before and after adjusting for potential confounders. Odds Ratios with their corresponding 95% confidence intervals (CI) were reported. Variables in the bivariate logistic regression with a *p*-value of less than 0.2 were fitted into the multivariable logistic regression to control the possible effects of confounders and identify the independent association between ANC and institutional delivery. Multicollinearity was checked using the variance inflation factor (VIF). According to a conservative threshold VIF value of 4, no collinearity was detected. In the multivariable analysis, variables with a *p*-value < 0.05 were considered as statistically significant.

## Results

### Socio-demographic and economic characteristics

Table 1 presents the descriptive statistics and status of the institutional delivery of the study sample. The mean (standard error) age of the mothers was 28.06 (0.14) years. Most (71.5%) were between the age of 20 to 34 years. Approximately, 38% of mothers were Orthodox Christian followed by Muslim (37.2%) and others (24.8%) were Catholic, Protestant or Traditional. Majority (87.3%) of mothers lived in rural areas. Moreover, 93.7, 53.4 and 21.8% of mothers were married, had no formal education and lived in the poorest wealth quintile, respectively. Nearly 20% of mothers were exposed to the media. More than half of women (58.1%), perceived the distance from nearby health facility as a big problem for utilization. Regarding wantedness, the most recent birth of the child, 74.3% of mothers reported that it was wanted then, and 17.9% were wanted later. More than a quarter (39.1%) of the women whose most recent childbirth was the fifth or above birth order. One-third (31.9%) of the mothers had four and above ANC follow-up while only, 11.3% of mothers gave the most recent birth in the health facility**.** Furthermore, institutional delivery significantly differed by maternal age, place of residence, education, marital status, exposure to the media, perceived distance from a nearby health facility, history of ANC follow-up, household wealth index, birth order and wantedness of the recent birth. A greater proportion of women who had four or more ANC visits delivered at a healthcare facility followed by women who had 1–3 ANC visits compared to women who had no ANC visits (21.9% vs. 10.5% vs. 2.9%; *p* < 0.001) (Table 1).

**Table 1:** Characteristics of study sample and the status of institutional delivery service utilization(*n* = 7575)

Variable	Number (%)	Institutional delivery		*P*-value^a^
		Yes Number (%)	No Number (%)	
Age				
13–19	8,35 (11.0)	84 (10.1)	751 (89.9)	< 0.001
20–34	5415 (71.5)	676 (12.5)	4739 (87.5)	
35–49	1325 (17.5)	97 (7.3)	1228 (92.7)	
Residence				
Urban	9,64 (12.7)	404 (41.8)	559 (58.2)	< 0.001
Rural	6611 (87.3)	453 (6.9)	6159 (93.1)	
Educational level				
No education	4780 (63.1)	264 (5.5)	4516 (94.3)	< 0.001
Primary	2147 (28.3)	290 (13.5)	1857 (86.5)	
Secondary or Higher	648 (8.6)	303 (46.8)	345 (53.2)	
Marital status				
Never married	56 (0.7)	17 (30.4)	39 (69.4)	0.004
Married	7097 (93.7)	779 (11.0)	6318 (89.0)	
Not currently married	422 (5.6)	362 (85.8)	60 (14.2)	
Exposed to media				
No exposed	6094 (80.4)	456 (7.5)	5638 (92.5)	< 0.001
Exposed	1481 (19.6)	401 (27.1)	1080 (72.9)	
Perceived distance from a nearby health facility				
Big problem	4402 (58.1)	295 (6.7)	4107 (93.3)	< 0.001
No big problem	3173 (41.9)	562 (17.7)	2611 (82.3)	
History of ANC follow up				
No	2818 (37.2)	82 (2.9)	2736 (97.1)	< 0.001
1–3	2342 (30.9)	246 (10.5)	2096 (89.5)	
4+	2415 (31.9)	529 (21.9)	1886 (78.1)	
Household wealth index				
Poorest	1648 (21.8)	60 (3.6)	1588 (96.4)	< 0.001
Poorer	1654 (21.8)	107 (6.5)	1547 (93.5)	
Middle	1585 (20.9)	1470 (92.7)	115 (7.3)	
Richer	1426 (18.8)	130 (9.1)	1296 (90.9)	
Richest	1262 (16.7)	444 (35.2)	818 (64.8)	
Birth order				
1	1431 (18.9)	311 (21.7)	1120 (78.3)	< 0.001
2–4	3183 (42.0)	371 (11.7)	2811 (88.3)	
5+	2961 (39.1)	176 (5.9)	2785 (94.1)	
Wantedness of the recent birth				
Wanted then	5563 (74.3)	671 (12.1)	4892 (87.9)	0.043
Wanted later	1317 (17.9)	128 (9.7)	1188 (90.7)	
Wanted no more	695 (9.2)	58 (8.3)	637 (91.7)	

^a^Rao-Scott chi-square *p*-value

Table 2 presents the crude and multivariable-adjusted association between ANC utilization and institutional delivery. In comparison with women with no antenatal care visit, the multivariable odds ratio (95% confidence interval) of institutional delivery among those who attend one to three and four or more antenatal care visit were 2.49 (1.66, 3.74) and 3.90 (2.60, 5.84), respectively. Other factors significantly associated with institutional delivery include urban residence 2.25 (1.44, 3.51), complete primary education 3.22 (2.09, 4.98), complete secondary or higher education 1.59(1.16, 2.17), poorer household wealth index 2.57 (1.57, 4.20), middle household wealth index 1.63(1.05, 2.52), richer household wealth index 1.56(1.03, 2,58) (Table 2).

**Table 2:** Association between antenatal care follow-up and institutional delivery service utilization(*n* = 7193)

Variable	Institutional Delivery	
	Unadjusted OR	Adjusted OR
Age		
13–19	1.42 (0.96,2.10)	0.76 (0.44,1.33)
20–34	1.81 (1.35,2.45)	1.10 (0.73,1.66)
35–49	Ref	Ref
Residence		
Urban	9.84 (6.98,13.86)	2.25 (1.44,3.51)**
Rural	Ref	Ref
Educational level		
No education	Ref	Ref
Primary	2.71 (2.05,3.50)	3.22 (2.09,4.98)**
Secondary and Higher	9.73 (6.51,14.01)	1.59 (1.16,2.17)*
Marital status		
Never married	2.64 (0.94,7.38)	1.66 (0.36,7.61)
Married	0.74 (0.52,1.06)	0.68 (0.44,1.07)
Currently not married	Ref	Ref
Exposed to media		
Yes	4.60 (3.52,6.02)	0.88 (0.63,1.23)
No	Ref	Ref
Perceived distance from health facility		
Big problem	Ref	Ref
No big problem	3.00 (2.30,3.90)	1.28 (0.98,1.67)
History of ANC follow up		
No	Ref	Ref
1–3	3.90 (2.60,5.85)	2.49 (1.66,3.74) **
4+	9.32 (6.30,13.80)	3.90 (2.60,5.84)**
Birth order		
1	4.39 (3.33,5.80)	1.47 (0.98,2.20)
2–4	2.09 (1.59,2.74)	0.98 (0.70,1.36)
5+	Ref	Ref
Household wealth index		
Poorest	Ref	Ref
Poorer	1.84 (1.19,2.85)	2.57 (1.57,4.20)**
Middle	2.08 (1.34,3.21)	1.63 (1.05,2.52)*
Richer	2.66 (1.71,4.13)	1.56 (1.03,2,58)*
Richest	14.41 (9.56,21.71)	1.6 (0.99,2.45)
Wantedness of the recent birth		
Wanted then	1.52 (1.04,2.25)	1.01 (0.66,1.55)
Wanted later	1.19 (0.75,1.91)	0.72 (0.44,1.74)
Wanted more	Ref	Ref

*p-value less than 0.005 **p-value less than 0.001

## Discussion

This study assessed the association between antenatal care follow-up and institutional delivery in Ethiopia. After adjusting age, distance from a nearby health facility, household wealth quintile, place of residence, exposure to media, birth order, educational level, and marital status, there was a significant positive association between antenatal care follow up and institutional delivery.

Compared to mothers who lived in rural areas, mothers who lived in urban areas were more likely to give birth in the health institution. This finding consistent with findings from other studies, including a recent systematic and meta-analysis in Ethiopia [10], EDHS 2016 [4], Further analysis of EDHS 2016 [11], Ghana [12] and India [13]. In the urban areas, because of the higher accessibility of maternal health services near their home and availability of transportation, women in urban areas are more likely utilized institutional delivery. Moreover, rural women are more affected by cultural taboos concerning the place of delivery than urban areas.

Women with higher levels of education have a greater awareness of sexual and reproductive rights and greater autonomy to decide on their own [12, 14]. This greater awareness may translate into more effective health-seeking behaviors and utilize hospital-based care. Furthermore, more educated women can communicate and understand information’s regarding institutional delivery, and identify danger signs easily. In this study, mothers who completed primary, secondary or higher education had higher odds of institutional delivery service utilization compared to mothers with lower educational attainment. This study supported by those of other studies from systematic and meta-analysis in Ethiopia [10], developing countries [15], Ghana [12] Uttarakhand [16], and India [17].

The odds of institutional delivery service utilization were higher among mothers with a poorer, middle, and richer wealth quintile compared to the poorest mothers. The finding was in agreement with the previous studies other developing countries [15], Ghana [12], Uttarakhand, and India [17]. The cost may help women to cover transportation and other expenses to bring and keep families at a health facility. Moreover, mothers who want to give birth in a private health institution needs to cover the cost of the service they need.

Finally, in this study, there was a significant association between antenatal care follow up and health facility delivery. The odds of institutional delivery service utilization higher among mothers who had ANC follow up one to three times and four or more times compared with mothers who had not ANC follow up during pregnancy. These results consistent with findings from other studies in Ethiopia [18], Nigeria [19], other African countries [20] and Nepal [21]. Antenatal care provides information for mother’s and her families on birth preparedness and complication. This can be an opportunity to promote the benefit of skilled attendance at birth and hospital-based delivery.

### Strength and limitations

The main strengths of the study are the use of large national probability samples and the availability of several confounding variables for adjustment in the multivariable regression model. Additionally, data were collected using a standardized questionnaire with rigorous procedures to check for data quality. However, as a cross-sectional study, the observed association between antenatal care use and institutional delivery should not be interpreted as causal. Additionally, this finding is may prone to recall and socially desirability bias because of exposure to media, distance from the nearby health facility and history of antenatal care data was collected based on self-reporting of participants.

## Conclusion

Increasing number of antenatal care use follow-ups was positively associated with institutional delivery service utilization. Thus, adherence to the recommended number of ANC follow up during pregnancy for all pregnant mothers is strongly recommended to improve the utilization of institutional delivery and prevent mothers and children from birth complications. Moreover, encouraging women through education and accessing maternal and child health services is recommended to increase institutional delivery service utilization.

## Data Availability

The datasets used for this study are publicly available from the DHS Program website http://dhsprogram.com/data.

## Abbreviations

ANC: Antenatal Care
AOR: Adjusted Odds Ratio
CI: Confidence Interval
DHS: Demographic and Health Survey
EDHS: Ethiopia Demographic and Health Survey
WHO: World Health Organization
